# The influence of dental occlusion on hamstring muscle isokinetic parameters in active competitive soccer athletes: a randomized controlled trial

**DOI:** 10.3389/fspor.2025.1589934

**Published:** 2025-07-23

**Authors:** Stine Hecht, Yoon Jeong Choi, Andrea Stroux, Florian Beuer, Manja von Stein-Lausnitz, Till E. Bechtold

**Affiliations:** ^1^ Department of Prosthodontics, Geriatric Dentistry and Craniomandibular Disorders, Campus Benjamin Franklin, Center for Dental and Craniofacial Sciences (CC3), Charité - Universitätsmedizin Berlin, corporate member of Freie Universität Berlin and Humboldt-Universität zu Berlin, Berlin Institute of Health, Berlin, Germany; ^2^ Department of Orthodontics, The Institute of Craniofacial Deformity, Yonsei University College of Dentistry, Seoul, Republic of Korea; ^3^ Institute for Biometrie and Clinical Epidemiology, Charité - Universitätsmedizin Berlin, Corporate Member of Freie Universität Berlin and Humboldt-Universität zu Berlin, Berlin, Germany

**Keywords:** dental occlusion, bite splint, muscle strength, isokinetic measurement, body performance, sports dentistry

## Abstract

**Introduction:**

The improvement of athletic performance depends on numerous factors including physical health, training status, neuromuscular coordination and psychological state. In recent years, the influence of dental occlusion and jaw position has gained increasing attention. The influence of occlusal bite splints (BS) on the improvement of motor performance, which is related to postural balance, has been the focus of many investigations. However, the extent of impact, that BS can have on muscle strength, has been attested to be unknown. Therefore, we wanted to investigate the influence of occlusion on the isokinetic strength parameters of the thigh muscles.

**Methods:**

Thirty active male competitive athletes at 18–32 years were included in this study. None had a gnathological or physiological disease. The subjects were randomly assigned to two experimental groups of 15 participants each, using a cross-over design. Concentric isokinetic muscle force measurements of the quadriceps and hamstring muscles were performed using a dynamometer under three occlusal conditions: habitual occlusion (HO), harmonic bite splint (HS), and simulated interference contact/malocclusion (MO). The three muscle force measurements were completed twice, each with one repetition at extension (E) and flexion (F) of the right knee, maximum force, and an angular velocity of 120°/s. Between the measurements, 120 s were scheduled for muscular recovery. The splint (HS) was 3D printed from a methacrylate-based resin and the harmonic occlusion was verified using the T-Scan Novus and occlusal paper. A unilateral punctual malocclusion (MO) made of light-curing methacrylate was individually placed in region 16. Statistical analysis was performed with one-factorial analysis of variance (ANOVA) followed by *post hoc* pairwise comparison.

**Results:**

There was a significant decrease in maximum torque (Nm) and muscle work (J) during extension movement with the simulated MO is compared to HO and to HS. Muscle power (W) was also decreased with MO compared to HO and HS. In the flexion movement, a significant decrease in muscle power was observed in the MO condition compared to the HS condition. No significance, but a tendency for improvement in extension and flexion movement in maximum torque, muscle work, and muscle power were determined in the HS condition compared in the HO condition.

**Conclusion:**

Our results indicate that simulated interfering occlusal contact can lead to a significant decrease and balanced occlusion to a tendency for improvement of isokinetic performance parameters in young, well-trained soccer players. This underlines a presumed importance of a balanced occlusion for muscle performance, especially in terms of maximum strength, muscle work and muscle power. The results show a functional relationship between dental occlusion and the lower extremities, which reflects an underlying anatomical connection in which the central nervous system may play a major role.

**Clinical Trial Registration:**

ClinicalTrials.gov, identifier, DRKS00030990

## 1 Introduction

Athletic performance is determined by a multitude of factors (1–3).

In recent years, growing attention has been paid to the impact of oral health on athletic performance in soccer players. Poor oral health has been linked to an increased risk of injury (4). Conversely, recent studies have also demonstrated that the stomatognathic system can positively influence athletic performance. Especially motor performance enhancement is a matter of interest in competitive sports and has been investigated in an extensive number of studies (5–7). As postural balance does contribute to optimizing motor performance (8–10), the influence of postural adaptions on athletic performance has also been observed in numerous investigations (11–13). In addition to that, the activity of distinct brain networks has been indicated to coordinate muscle synergies during functional tasks (14), which further suggests investigation of a possible relation between mandibular balance or position and certain athletic performance tasks.

From the late 1970s on, scientific study results have favored the premise that jaw posture repositioning can enhance appendage muscular strength and athletic performance (15). Alteration of occlusal contacts or of mandibular position can be achieved, using occlusal splints (OS) or custom-fabricated mouthguards (MG), which block and balance bite contacts between upper and lower dentition. In terms of improvement of exercise performance, dental occlusion and jaw position have received increased attention during the last years (16–18).

A study with 25 female soccer players investigated and confirmed the effect of various MG on athletic performance (19). Applications of OS have been used to guide the mandibular condyles in a centric position within their temporomandibular joints (TMJs) which led to increased shoulder strength in 20- to 22-year-old men (20). In another investigation in amateur rugby players at the age of 21–29, mouthguards (MG) were used to achieve a congenial effect of centric TMJ relation, which led to enhanced peak force and peak acceleration in ballistic bench press (21).

On the other hand, occlusion has been manipulated using custom-fabricated, bilaterally balanced, dual-laminated MGs during 75% maximum power clean lifts, performed by 24 healthy weightlifters, which was leading to a self-perception of being stronger and less encumbered (22). In another study, 12 healthy female dental students experienced a significant decrease in knee eccentric muscle performance when their occlusion was imbalanced, as compared to balanced occlusal condition using OS (23).

However, the extent of impact, that occlusal splints (OS) can have on muscle strength, has been attested to be unknown (24). In order to elucidate possible changes that may result from OS during exercise, more studies on the specific topic—using simple and easily reproducible experimental designs—are needed for drawing more definite conclusions (24). Therefore, in this study we aimed to investigate the influence of dental occlusion on the isokinetic strength parameters of the femoral musculature.

We hypothesized that different occlusal conditions will not lead to different isokinetic parameters of the thigh muscles.

## 2 Materials and methods

The study had been approved by the Ethics Commission of Charité—Universitätsmedizin Berlin (EA4/152/22) and registered in the German Register of Clinical Studies (DRKS). In advance, all subjects had given their written informed consent for participating, using, and publishing the collected data. The study was conducted according to ethical standards in the Declaration of Helsinki from 1964. The 1997 Good Clinical Practice Recommendation was adhered to.

### 2.1 Participants

**Figure 1:**
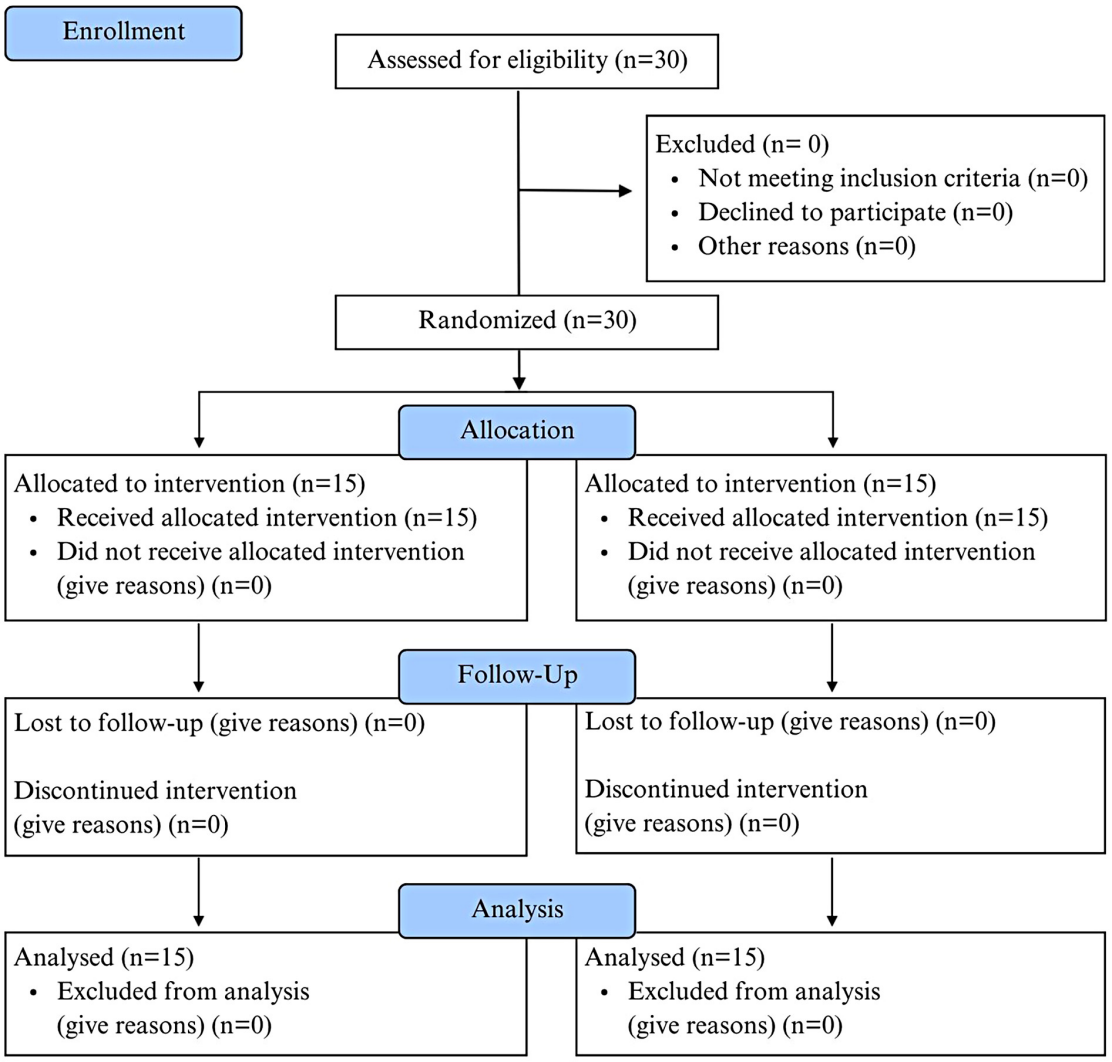
Consort 2010 flow diagram.

The recruitment period for this study was from January 2023 to March 2023. Thirty active male competitive soccer athletes (of the same professional soccer club), at 18–32 years of age, were included in the study. All had a similar physical fitness status due to an equal training load and intensity (5–7 days/week) at the same soccer training center. Exclusion criteria were an existing injury in the knee and thigh area and operations in the head and neck area less than three months ago. None of the subjects had to be excluded from the study for any of these reasons (Figure 1). In addition, only subjects who had no evidence of craniomandibular dysfunction were included.

The gnathological examination was carried out in accordance with the CMD screening (CMD basic diagnostics) of the German Society for Functional Diagnostics and Therapy (DGFDT).

### 2.2 Experimental design

This prospective study was performed with a cross-over design of two experimental groups. Thirty subjects were randomly assigned to two groups of 15 participants each. For randomization, subjects were numbered consecutively in random order and assigned based on the numbers. The sample size calculation showed that with a case number of *n* = 30, a mean difference of 25.5 Nm (122.5 Nm condition 1 vs. 97 Nm conditions 2 and 3) with a standard deviation of 39 can be statistically proven with 80% power using linked *t*-tests at a global two-sided significance level of 5% (1.25% local significance level after Bonferroni correction). The case number estimation was carried out using the software nQuery + nTerim 4.0.

#### 2.2.1 Preparation of simulated occlusion

For each subject, we fabricated an individual harmonic occlusal bite splint (BS) in the maxilla and an attachable interference contact to simulate different bite situations.

Based on an intraoral 3D scan, using a Primescan 2 (Dentsply Sirona Inc., Charlotte, NC, USA), an individual BS was digitally designed to achieve a harmonious occlusion. Arbitrary digital articulation was utilized to block the bite in a position of 4 mm elevation at the incisors. This allowed for an equal and reproducible situation in each subject.

Subsequently, the BS were 3D printed from a methacrylate-based resin. An interference contact was fabricated in region 16 from light-curing methyl methacrylate and peroxide-free composite, which could be individually attached to the BS. This simulated a unilateral punctual malocclusion of 3 mm.

**Figure 2:**
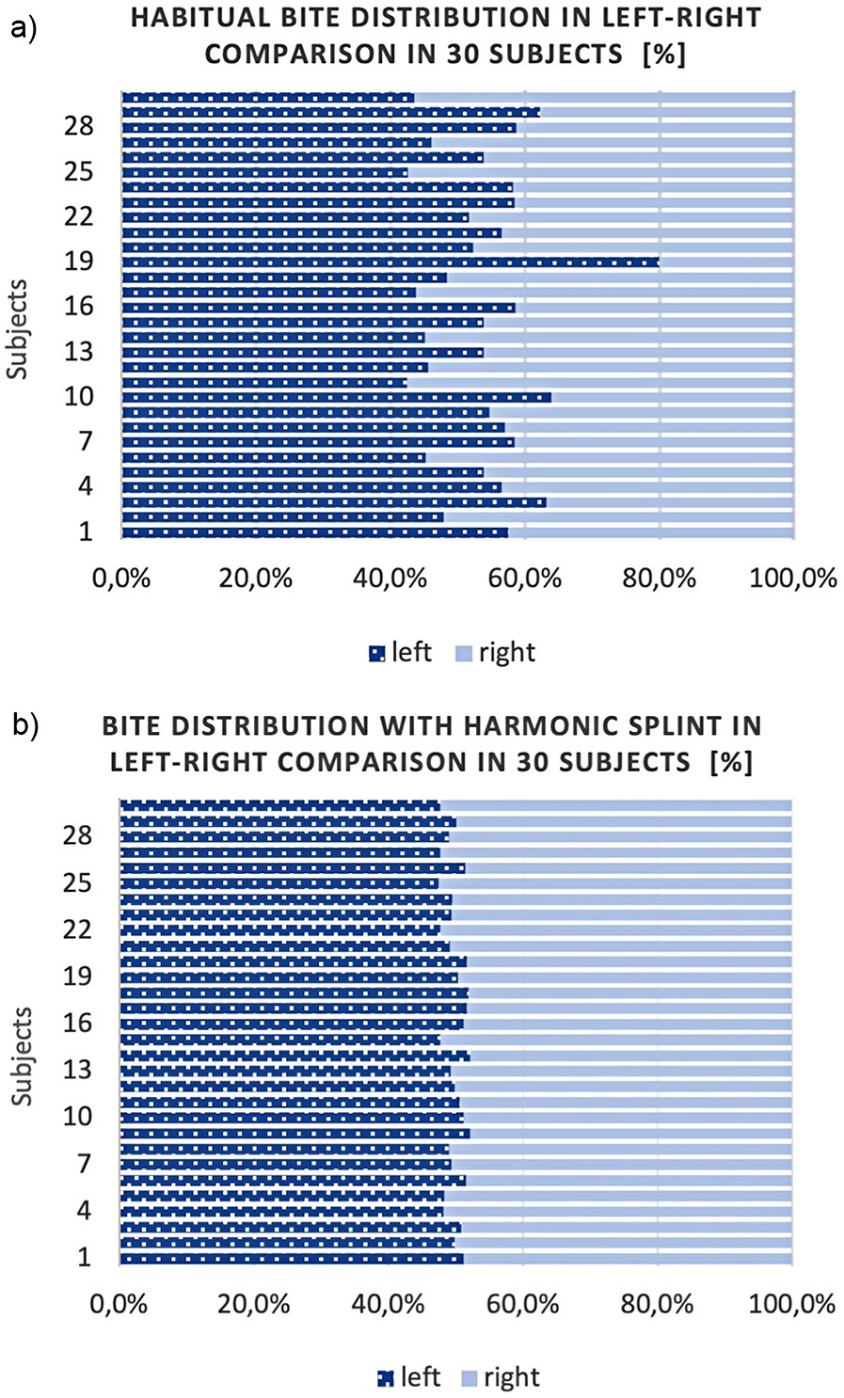
Shows the bite of the 30 subjects in left/right comparison with **(a)** habitual occlusion and **(b)** harmonic bite splint in %.

**Figure 3:**
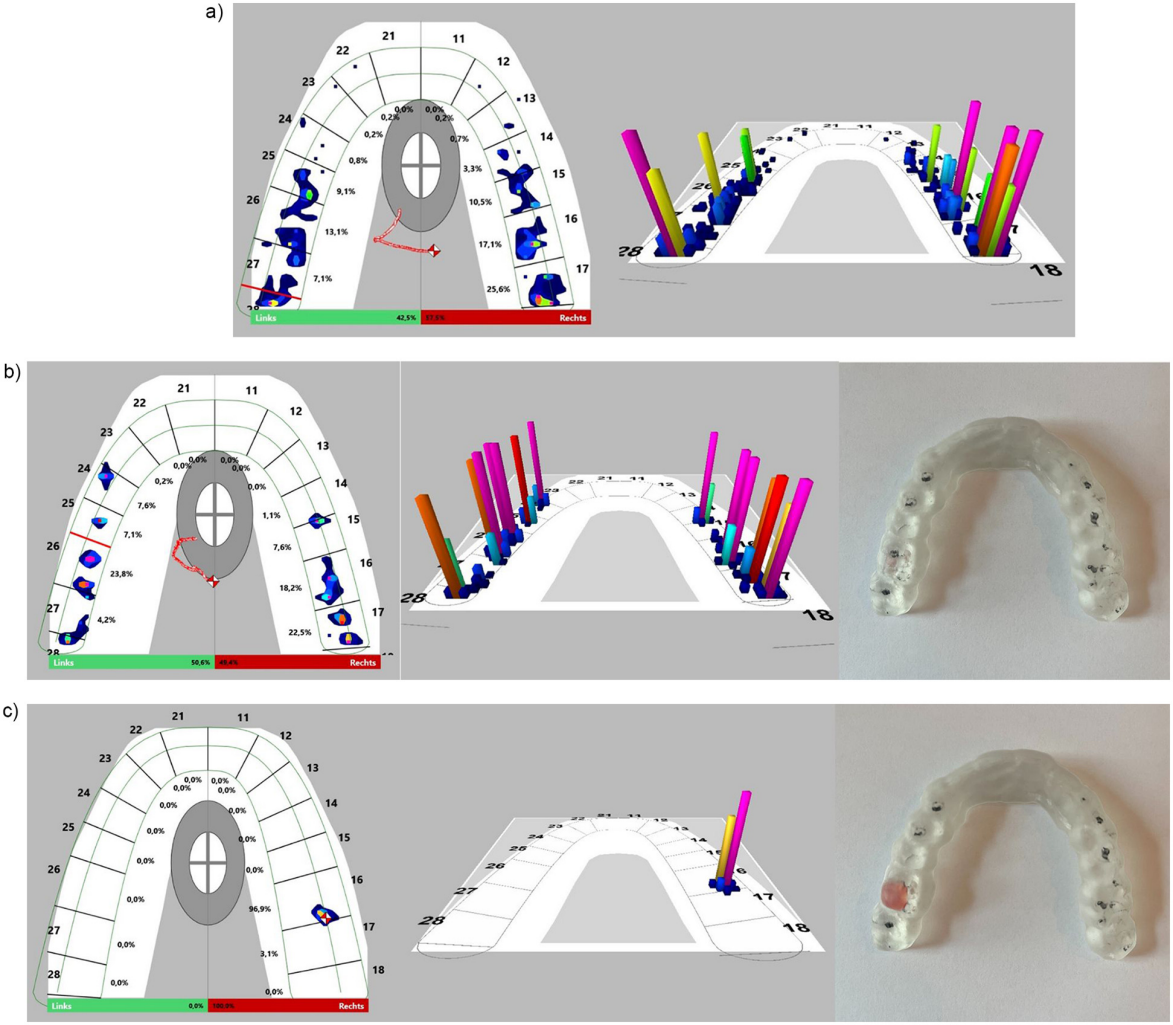
Shows the software analysis of the T-scan Novus based on a subject example with **(a)** habitual occlusion (left/right 42.5%/57.5%), **(b)** harmonic bite splint (left/right 50.6%/49.4%), **(c)** malocclusion/ dental interference contact on the right (left/right 0%/100%).

Intraorally, the contact point distribution of the habitual occlusion at maximum intercuspation (MI) was displayed as a percentage in a left/right comparison using T-Scan Novus and digital software. As a comparison of the contact point distribution with and without BS, the occlusion analysis was also performed with the BS in MI. An occlusion with uniform contact point distribution and a left/right aspect ratio of 50%/50% (max 5% diff.) was set on the BS. In addition, the occlusion was verified with occlusal paper (Figures 2,3).

#### 2.2.2 Testing procedure

The subsequent muscle force measurements were performed on an isokinetic force gauge (BIODEX®). Three concentric muscle force measurements were completed twice, each with one repetition at extension (E) and flexion (F) of the right knee, maximum force, and an angular velocity of 120°/s. The muscle force measurements were performed in three different occlusal situations. Between the six measurements, 120 s were scheduled for muscular recovery.

The first experimental group performed the first measurement in a habitual occlusal (HO) without a splint in habitual MI, the second with harmonic BS in MI (HS), and the third with the interference contact attached, representing malocclusion (MO).

The order of harmonic BS and interference contact was switched in the second experimental group. The first measurement was performed with HO, the second with MO, and the third with HS.

**Figure 4:**
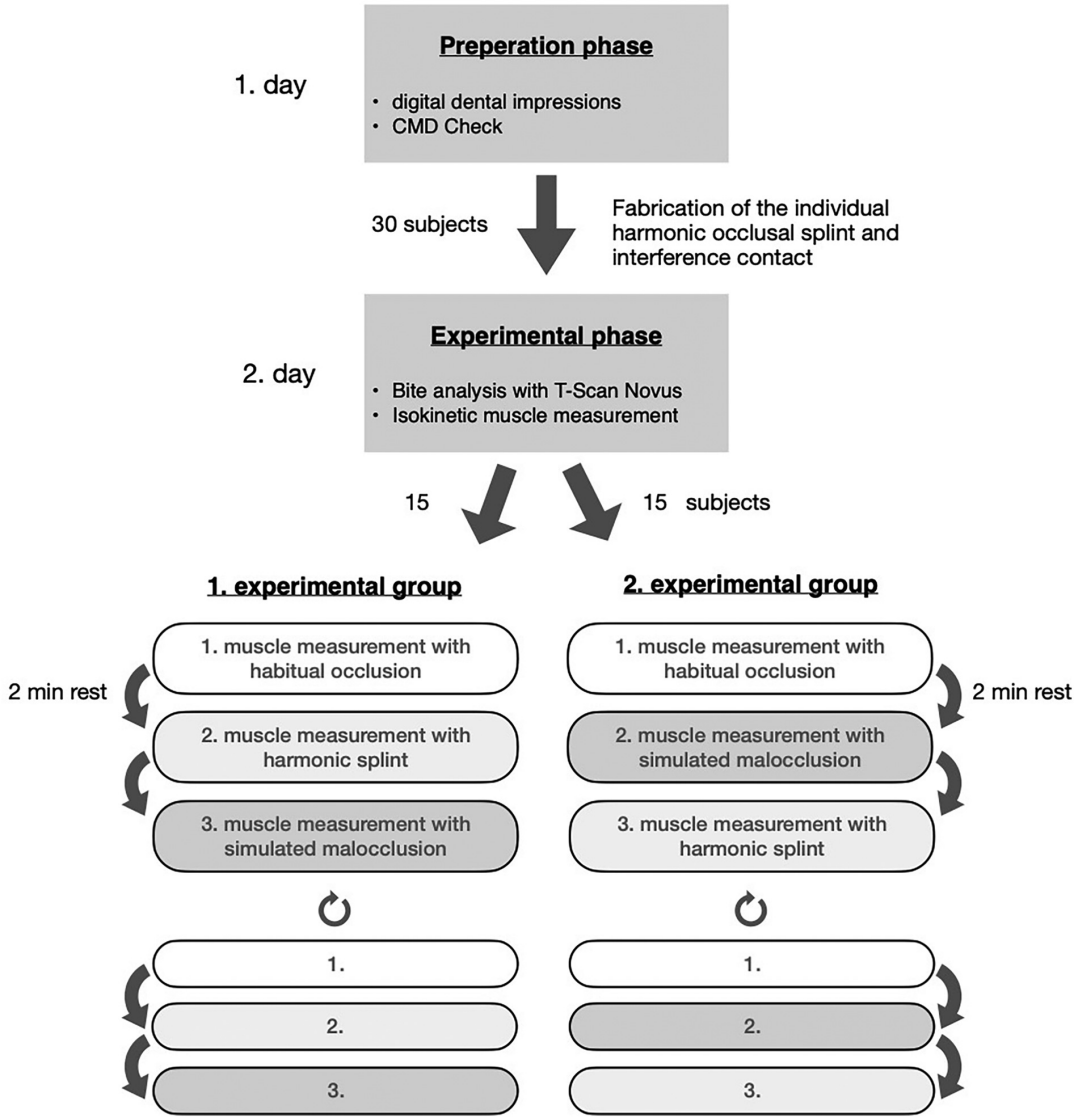
Scheme of the experimental procedure for the 2 days of the study. 1st day (preparatory phase), 2nd day (experimental phase). The subjects were randomly assigned to the two groups.

Subsequently, the three runs were repeated for both groups to exclude measurement errors due to a learning effect when performed for the first time. Accordingly, only the second repetition of the three measurements was included in the data analysis (Figure 4).

#### 2.2.3 Analysis of measurement data

After the measurements, the software of the isokinetic force gauge compiled a complex analysis showing the main isokinetic parameters such as maximum torque (M) [Nm], total work (W) [J], and maximum power (P) [Watt].

**Figure 5:**
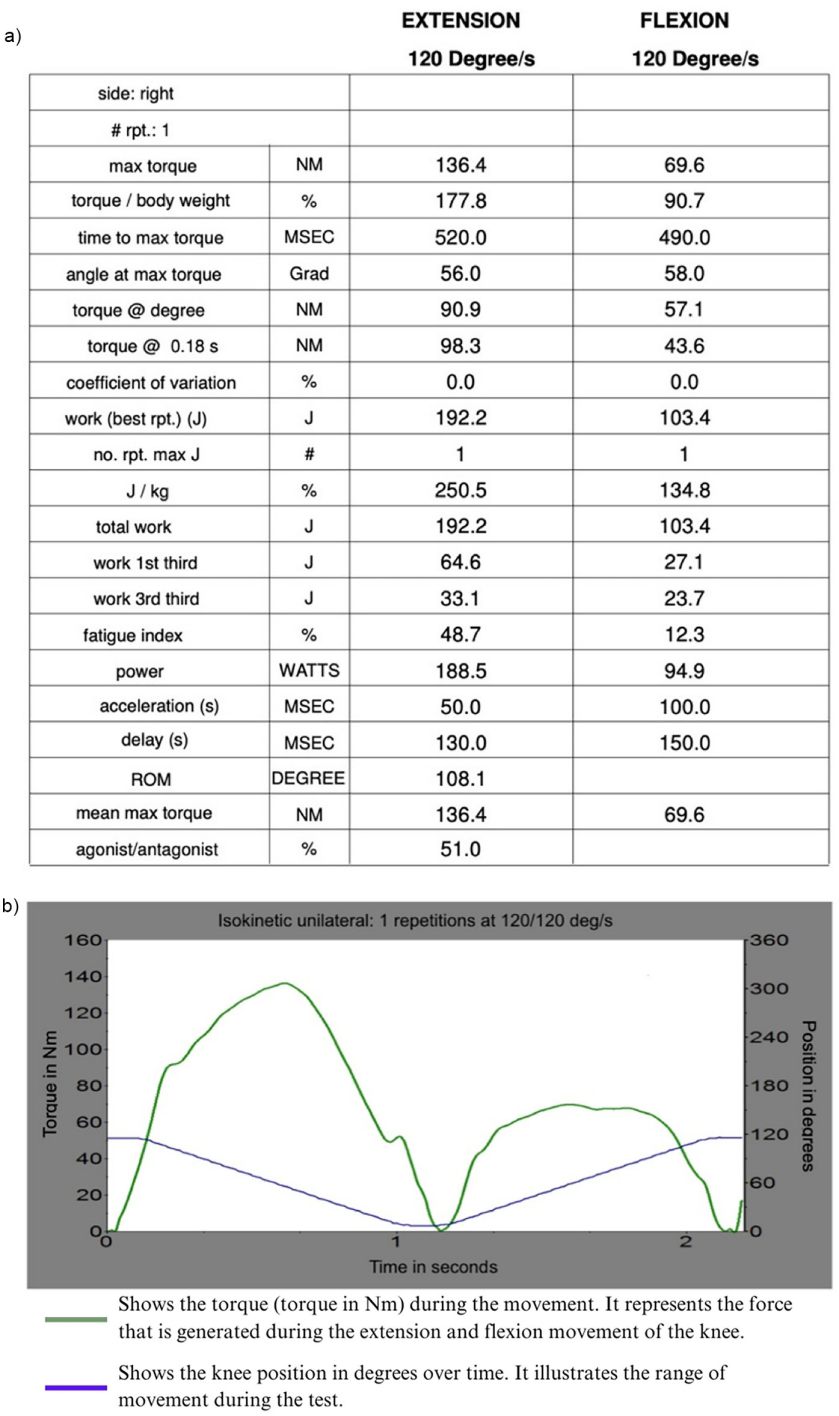
Shows **(a)** an example of a complex analysis of the isokinetic force gauge and **(b)** the corresponding torque curve and range of movement during extension and flexion of the right knee.

The parameters were recorded for each, the extension and the flexion movements, in order to provide information on the knee extensor muscle (quadriceps femoris) during extension (E) and on the knee flexor muscle (hamstring) during flexion (F) (Figure 5).

The values of these parameters were then analyzed, comparing the different bite situations.

The maximum torque (M) reflects the maximum force development achieved within the repetition and is, therefore, a correlate of the maximum force. Total work (W) is expressed in joules [J] and describes the total muscular force development over the complete range of motion (ROM). Physically, work (W) is the product of force (F) and displacement (s). In terms of isokinetic dynamometry, the work is calculated from the measured torque multiplied by the ROM. It can be derived as an integral of the area under the torque curve.

**Figure 6:**
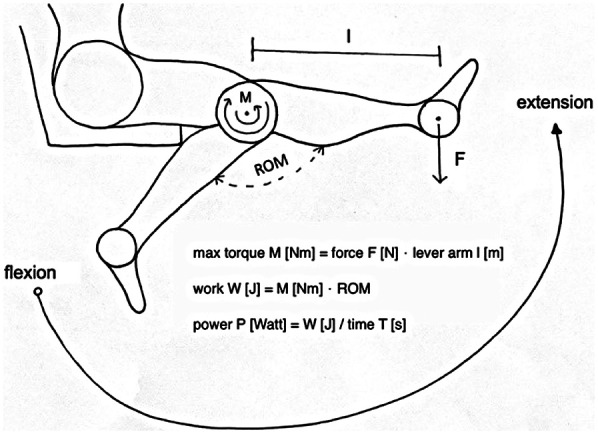
Shows the movement execution during the force measurement with the resulting isokinetic parameter.

Power (P), on the other hand, corresponds to the total work (W) of the best repetition or the total of all repetitions per unit time (T) and is expressed in watts (Figure 6) (25, 26).

### 2.3 Statistical analysis

For the description of qualitative variables with ordinal or nominal scale level, absolute and percentage frequencies were given. The continuous measurement variables examined here were descriptively presented using the mean with standard deviation, the minimum and maximum, and the median with first and third quartiles (interquartile range), respectively. To test whether there was a significant deviation from a normal distribution, the Kolmogorov–Smirnov test was applied. Since no significant deviations from a normal distribution were detectable, parametric procedures could be used for further analysis. Thus, analysis of variance for repeated measures was used to test whether significant differences could be detected in the three primary outcome measures: max torque, performance, and work between the situations “habitual,” “harmonic,” and “interference contact.” If this was the case, the global test was followed by a Bonferroni-corrected *post hoc t*-test pairwise comparison to examine which bite block situations differed from each other pairwise. Boxplots were used to visualize the measurement results.

All tests are two-sided, and a significance level of 5% was used. IBM SPSS Statistics 28 (SPSS Inc. an IBM Company, Chicago, IL) was used to perform the biometric analyses.

## 3 Results

The collected data were grouped according to the influence of the three bite situations: HO, HS, and MO on maximum torque (M), power (P), and work (W) in extension (E) and flexion (F).

### 3.1 Effect of dental occlusion on the maximum torque (M) [Nm]

#### 3.1.1 Extension

**Figure 7:**
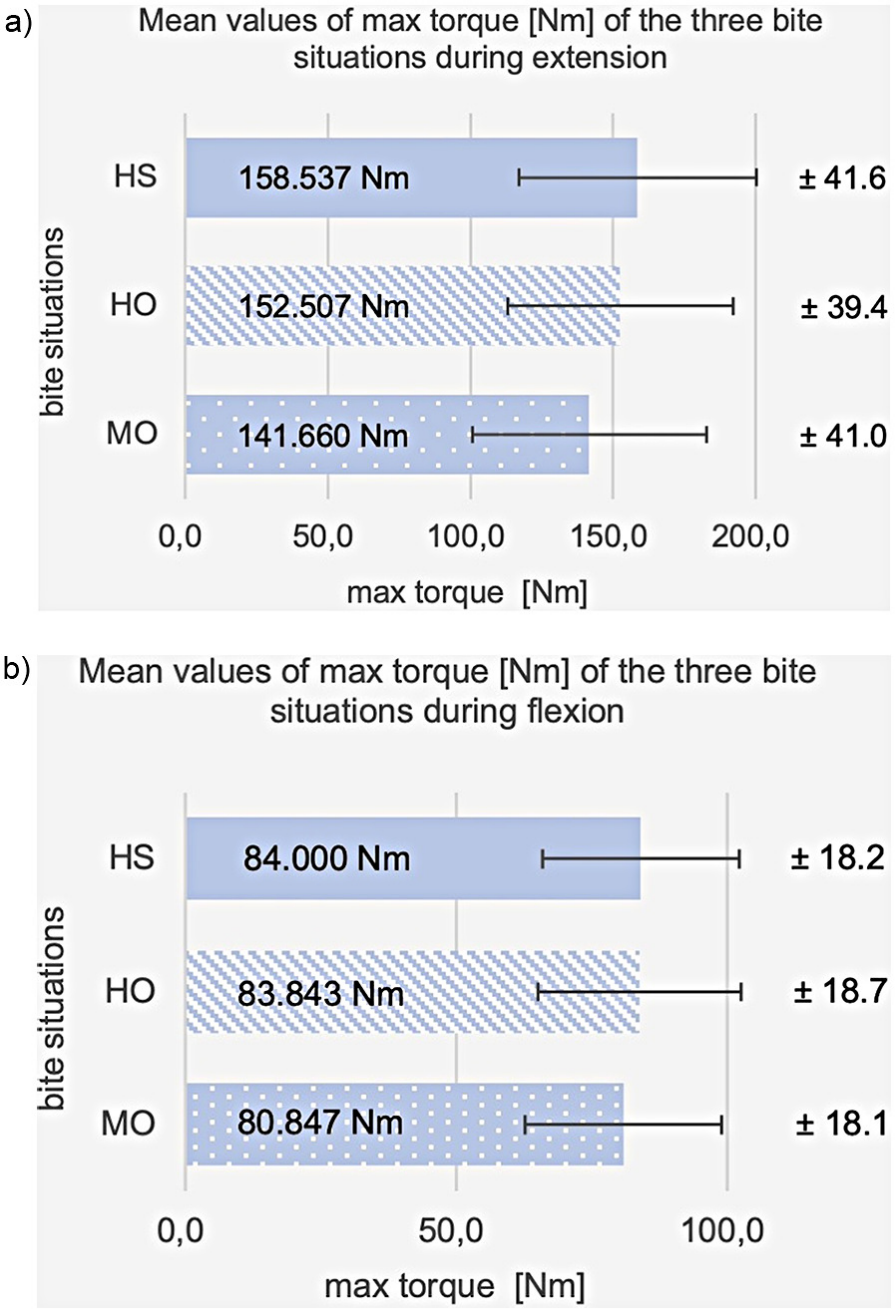
Shows a cross-bar graph representing the average values and standard deviations (SD) of the max. torque at habitual occlusion (HO), harmonic bite splint (HS), and malocclusion (MO) **(a)** during extension, **(b)** during flexion.

The max torque [in Nm] showed significant differences during extension of the knee between the three bite situations: HO, HS, and MO [F(2.3) = 17.5; *p* < 0.001]. There was a significant difference in max torque during extension between HO and MO in the *post hoc* pairwise comparison (*p* = 0.002) and between HS and MO (*p* < 0.001). In contrast, the max torque of HO was not significantly different from that of HS (*p* = 0.137). The average value of max torque in HO is 152.5 Nm (SD = 39.5), lower than in HS 158.5 Nm (SD = 41.6) and higher than in MO 141.7 Nm (SD = 41.0), see Figure 7. In extension movement, compared to habitual initial occlusion, an average percentage decrease in max torque in MO of −7.3% and an average percentage increase in max torque in HS of +4.4% were obtained.

#### 3.1.2 Flexion

On flexion, a significant difference in max torque was found between the three bite situations [F(2.3) = 3.5; *p* = 0.038]. In the pairwise comparison, no differences are detectable after Bonferroni correction (*t*-test for connected samples, *p* ≥ 0.05). The mean value of max torque for HO is 83.8 Nm (SD = 18.7), for HS 84.0 Nm (SD = 18.2), and for MO 80.8 Nm (SD = 18.1), see Figure 7. The percentage difference from the HO is +1.0% for HS and −3.3% for MO.

The measurement results of the second measurement round during extension and flexion are listed in Table 1.

**Table 1:** Shows the measurement results of max. torque of the second measurement round.

Measurement results of max. torque in [Nm]						
Subjects	HO		HS		MO	
	E	F	E	F	E	F
1	109	48,7	100,6	49,4	81,4	44,3
2	164,7	107	181,9	110,3	158,6	109,7
3	131	96,8	149,3	94,3	96,2	94,5
4	118,7	88	119	92,4	118,2	88,7
5	130,6	68	129,1	70	108,4	60,9
6	165,8	71,2	146,9	64,9	136,8	71,2
7	184,4	117,8	174,6	115,2	159	112,3
8	140,1	65,1	158,9	69,2	134,1	61,8
9	120,6	56,1	102,4	66,8	118,3	67,7
10	102,3	60,4	95,3	67,8	84,7	55,4
11	51,8	72,1	54,6	68	56,2	65,7
12	220,2	82,6	240,4	88	227,2	83,5
13	169,4	100,7	166,1	93,6	171,9	95,1
14	182,8	98,9	198,3	92,5	184,2	93,2
15	167,7	97,6	213,7	99,4	166,7	89,5
16	170,2	98,1	141,9	88,5	171,7	95,1
17	95,3	76,1	113,8	89,4	137,2	87,5
18	122,2	70,7	133,1	66,2	140,3	60,5
19	164,7	96,4	157	88,8	171,7	89,6
20	149,7	58,8	119,9	60,2	170,5	78,9
21	136,6	84,9	115,6	75,9	140	76
22	171,8	76,6	171,1	78,9	189	83,2
23	213,2	110,8	209,3	111	206,8	108,4
24	209,8	112,2	201	108,4	208,5	125,1
25	163,1	78,3	157,9	76,3	164,9	86,9
26	98,9	63,6	77,3	60,1	98,1	69,4
27	207,6	100	178,8	78,8	190,6	83,3
28	180,4	104,1	186,1	98	198,6	102,9
29	150,1	69,9	149,2	61	160,4	56,3
30	182,5	83,8	135,9	90,4	176,7	75,1

HO, habitual occlusion; HS, harmonic bite splint; MO, malocclusion; E, extension; F, flexion.

### 3.2 Effect of dental occlusion on power (P) [watts]

#### 3.2.1 Extension

**Figure 8:**
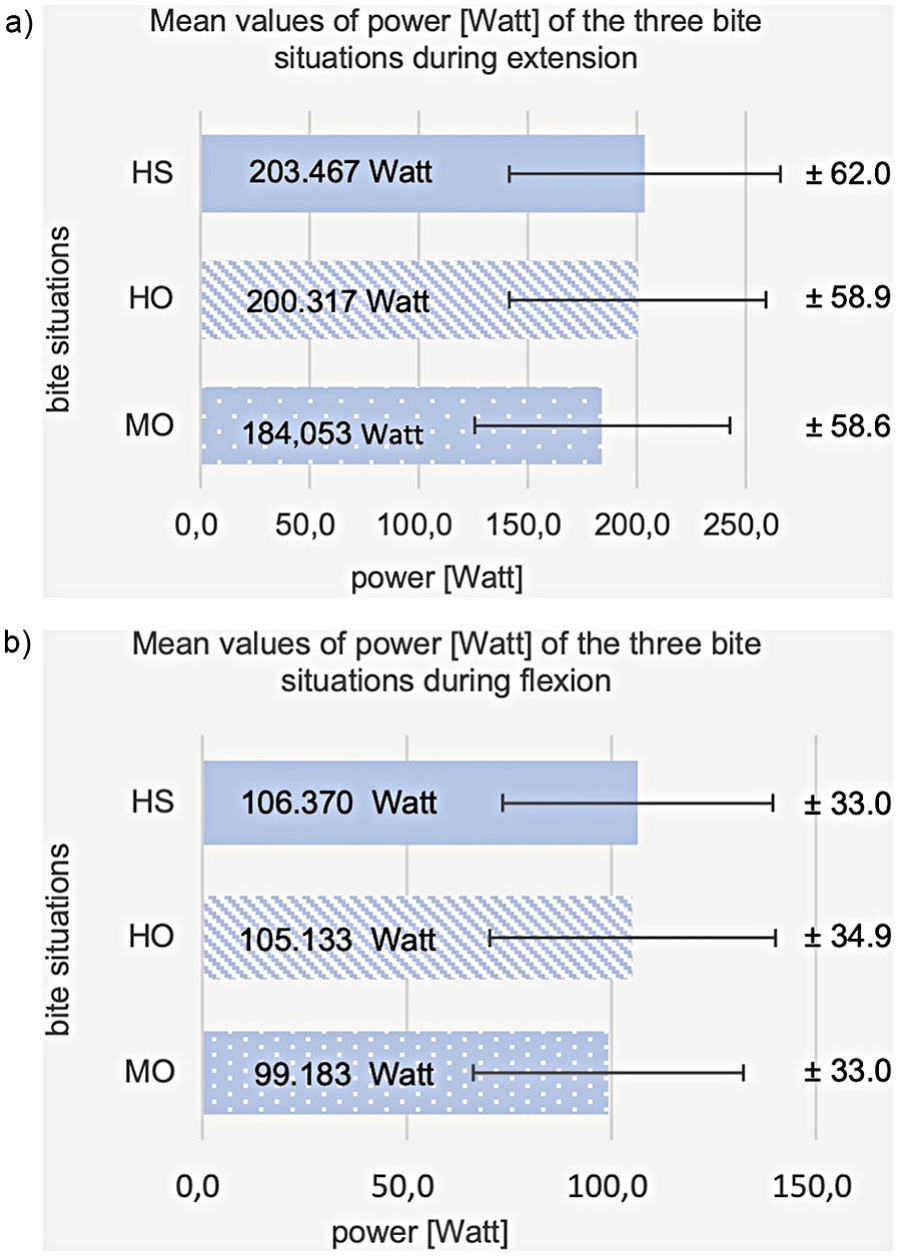
Shows a cross-bar graph representing the average values and standard deviations (SD) of the power at habitual occlusion (HO), harmonic bite splint (HS), and malocclusion (MO) **(a)** during extension, **(b)** during flexion.

Power (P) showed significant differences during knee extension between HO, HS, and MO [F (2.3) = 13.7; *p* < 0.001]. The pairwise comparison showed a significant difference between HO and MO (*t*-test for connected samples with Bonferroni correction, *p* = 0.001) and between HS and MO (*p* < 0.001). *P* is insignificant between HO and HS (*p* > 0.999). The mean value at extension is 200.3 watts (SD = 58.9) for HO, 203.5 watts (SD = 62.0) for HS, and 184.1 (SD = 58.6) watts for MO, see Figure 8. In the percentage difference from HO, *P* increased by +2.0% for HS and decreased by −7.8% for MO.

#### 3.2.2 Flexion

*P* at flexion significantly differed between all three bite conditions [F(2.3) = 3.5; *p* = 0.036]. A significant difference was still detected in the pairwise comparison between HS and MO (*t*-test for connected samples with Bonferroni correction *p* = 0.041). There is no significance between HO and MO (*p* = 0.231) or HO and HS (*p* > 0.999). The mean value is 105.1 watts (SD = 34.9) for HO, 106.4 watts (SD = 33.0) for HS, and 99.2 watts (SD = 33.0) for MO, see Figure 8. The percentage difference from HO is +3.2% for HS and −3.5% for MO.

### 3.3 Effect of dental occlusion on work (Wo) [J]

#### 3.3.1 Extension

**Figure 9:**
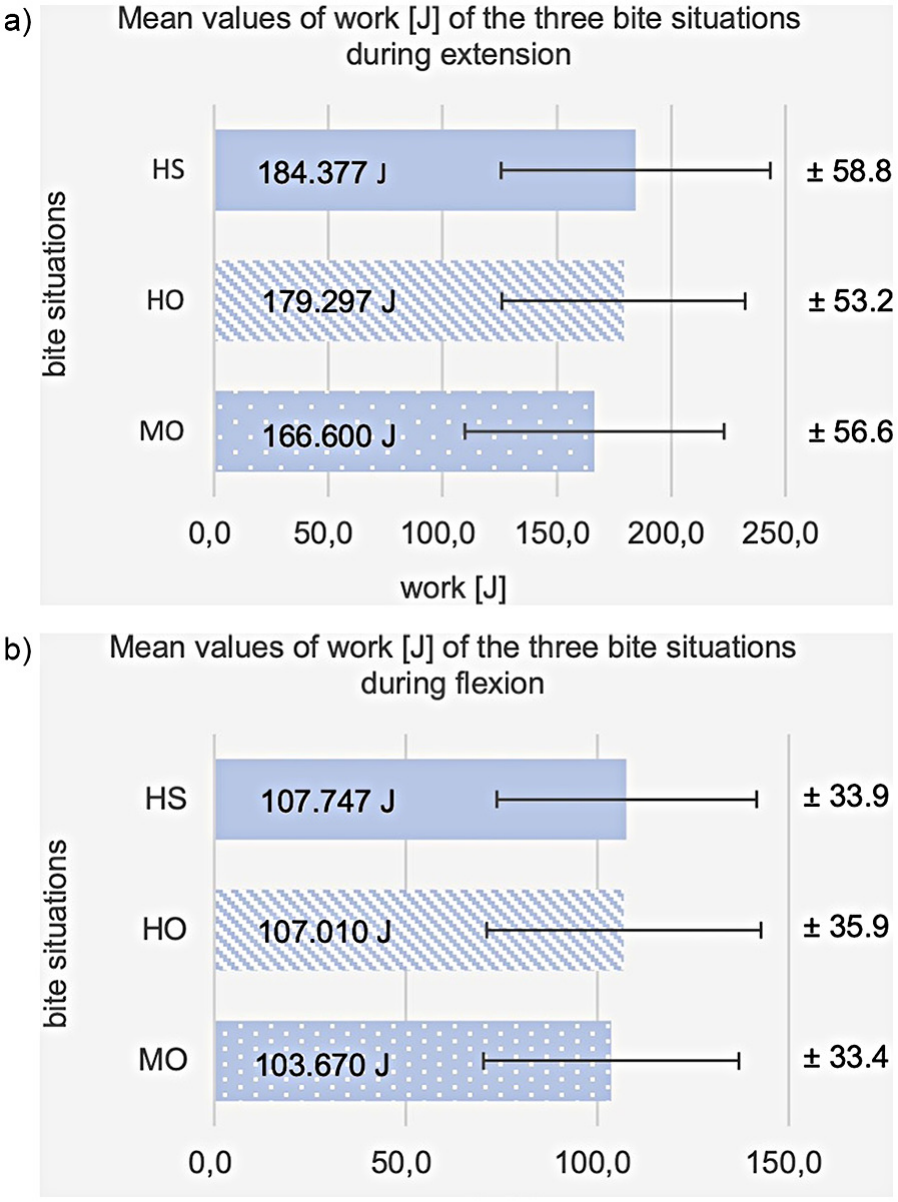
Shows a cross-bar graph representing the average values and standard deviations (SD) of the work at habitual occlusion (HO), harmonic bite splint (HS), and malocclusion (MO) **(a)** during extension, **(b)** during flexion.

Work (Wo) showed equally significant differences during extension of the knee between the three bite situations: HO, HS, and MO [F(2.3) = 14.5; *p* < 0.001]. *post hoc* pairwise comparison showed a significant difference in Wo during extension between HO and MO (*t*-test for connected samples with Bonferroni correction, *p* = 0.002) and between HS and MO (*p* < 0.001). In contrast, HO's work is not significantly different from HS's (*p* = 0.495). The average value of work in HO is 179.3 J (SD = 53.2), which is lower than in HS 184.4 J (SD = 58.8) and higher than in MO 166.6 J (SD = 56.6), see Figure 9. In extension movement, compared to HO, an average percentage decrease in work in MO of −7.7% and an average percentage increase in work in HS of +2.8% was obtained.

#### 3.3.2 Flexion

**Figure 10:**
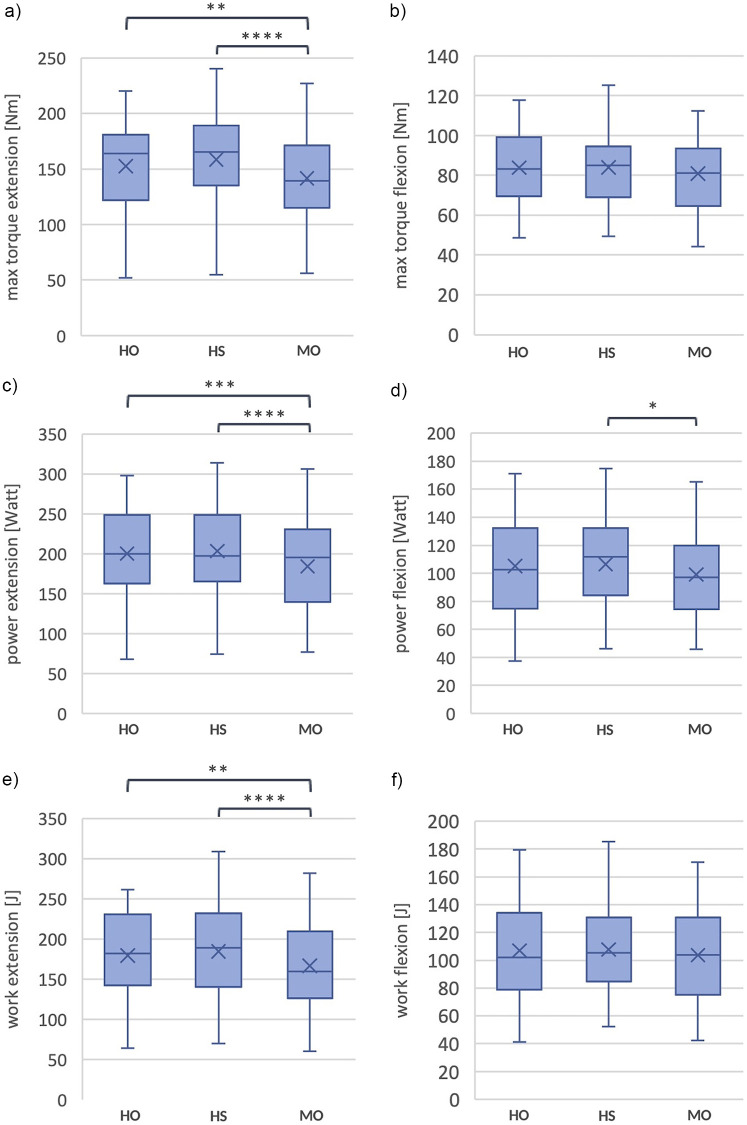
Shows box plots with comparison between habitual occlusion (HO), harmonic bite splint (HS) and malocclusion (MO) of **(a)** max. torque during extension, **(b)** max. torque during flexion, **(c)** power during extension, **(d)** power during flexion, **(e)** work during extension, **(f)** work during flexion. The box plots with frames representing the 1st and 3rd quartiles and the median demonstrating the thick bar within this box. The “X” mark shows the mean value. The upper and lower whiskers illustrate the minimum and maximum. *: *p* = 0.041, **: *p* = 0.002, ***: = *p* = 0.001, ****: *p* = <0.001.

No significant difference in work between the three bite situations was found for flexion [F(2.27) = 2.5; *p* = 0.094]. The mean value of work for HO was 107.0 J (SD = 35.9), for HS 107.7 J (SD = 33.9), and MO 103.7 J (SD = 33.4), see Figure 9. The percent difference from the HO is +2.3% for HS and −2.3% for MO. Figure 10 shows a comparison of the statistical parameters of maximum torque, power, and work during the three biting situations. Table 2 shows an overview of the results comparing the three occlusal conditions in terms of maximum torque, power, and work during extension and flexion.

**Table 2:** Shows an overview of the results comparing the three occlusal conditions [habitual occlusion (HO), harmonic bite splint (HS), and malocclusion (MO)] in terms of maximum torque, power, and work during extension and flexion.

Isokinetic parameters	Movement	Occlusal conditions		
		HS	MO	HO
Maximum torque (M) [Nm] (Standarddeviation; percentage difference from HO)	Extension	158.5 (SD = 41.6; + 4.4%)	141.7 (SD = 41.0; −7.3%)	152.5 (SD = 39.5)
		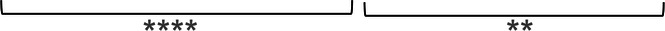		
	Flexion	84.0 (SD = 18.2; + 1.0%)	80.8 (SD = 18.1; −3.3%)	83.8 (SD = 18.7)
Power (P) [Watts] (Standarddeviation; percentage difference from HO)	Extension	203.5 (SD = 62.0; + 2.0%)	184.1 (SD = 58.6; −7.8%)	200.3 (SD = 58.9)
		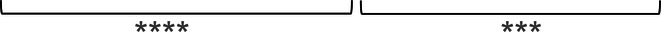		
	Flexion	106.4 (SD = 33.0; + 3.2%)	99.2 (SD = 33.0; −3.5%)	105.1 (SD = 34,9)
		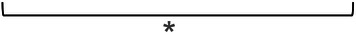		
Work (Wo) [J] (Standarddeviation; percentage difference from HO)	Extension	184.4 (SD = 58.8; + 2.8%)	166.6 (SD = 56.6; −7.7%)	179.3 (SD = 53.2)
		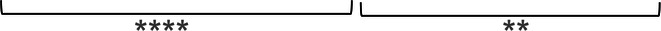		
	Flexion	107.7 (SD = 33.9; + 2.3%)	103.7 (SD = 33.4; −2.3%)	107.0 (SD = 35.9)

Significance levels: *: *p* = 0.041, **: *p* = 0.002, ***: *p* = 0.001, ****: *p* < 0.001.

## 4 Discussion

The present study aimed to determine the effects of different dental occlusion conditions (HO = habitual occlusion, HS = harmonic splint, MO = malocclusion) on (i) the maximum torque, (ii) the muscle work, and (iii) the muscle performance of the knee extensor and flexor muscles during extension and flexion of the right leg. A homogeneous group of young adult competitive soccer players was included in the study.

The main findings were the following: (i) there was a significant decrease in maximum torque (Nm) and (ii) muscle work (J) during extension movement when a simulated MO is set compared to HO (*p* = 0.002) and to HS (*p* < 0.001). (iii) Muscle power (watts) also decreased with MO compared to HO (*p* = 0.001) and HS (*p* < 0.001). In the flexion movement, a significant decrease in muscle power was observed in the MO condition compared to the HS condition. No significance, but a tendency for improvement in extension and flexion movement in maximum torque, muscle work, and muscle power were determined in the HS condition compared with the HO condition.

Our results indicate that simulated interfering contact can lead to a significant decrease and balanced occlusion to a tendency for improvement of isokinetic performance parameters. These findings are fairly consistent with the results of Grosdent et al. (23), who used a similar study methodology to create an unbalanced occlusion using a 1-mm-thick piece of resin. The imbalanced occlusion corresponded to an average decrease of 9% in eccentric peak torque (23).

Another study confirms the negative influence of simulated malocclusion on the muscle strength of young competitive rowers (27). The negative influence of simulated malocclusions on the maximum torque, muscle work, and muscle power of the right knee extensor and flexor refutes our hypothesis that different occlusal conditions will not lead to different isokinetic parameters of the thigh muscles.

The relationship between malocclusion and physiological effects on the body has already been investigated in several studies. It has been shown that an erect position of the head is maintained by a tension balanced between nociceptive stimuli originating from the glossopharyngeal and vagal nerve and/or from neck and dural vessels within the trigeminal subnucleus caudalis (28, 29). Trigeminal afferents have been shown to influence posture in a study where the anesthesia of its mandibular branch modified postural control in human subjects (30). This indicates a direct adaptation of the body to manipulation of the stomatognathic system.

Hence, the functional and anatomical relationships between the masticatory system and posture control system give the rationale for a possible association between postural control and malocclusions (31).

In comparison with other studies, a distinction is made as to whether the malocclusion was experimentally simulated or occurred naturally without manipulation. Studies that investigated the influence of natural malocclusion showed fewer clear results on physical performance parameters than studies with manipulated malocclusion. Natural malocclusion showed no clear influence on maximal aerobic capacity, athletic performance (32), and neuromuscular control (33). Another study showed that natural malocclusion characteristics have no negative influence on balance ability (34).

The Results show that simulated malocclusion may not reflect natural clinical conditions, which develop over longer periods of time and can be accompanied by compensatory neuromuscular adaptations. Therefore, the generalizability of our results to individuals with natural malocclusion is limited.

Nevertheless, simulated malocclusion could also cause a short-term neuromuscular adaptation, known as motor learning. In isokinetic tests, the learning effect refers to an improvement in performance resulting from practice or experience rather than an actual increase in strength. This means that with repeated attempts, participants become more familiar with the task and can perform their movements more efficiently, leading to an increase in strength values (35). For this reason, we did not include the values from the first measurement round in the analysis, as the greatest learning effect was evident from the first measurement to the second.

In order to draw meaningful conclusions, further studies should include a special familiarization phase before the test sessions to further minimize possible bias due to motor adaptation. This would allow comparisons between simulated and natural malocclusion to be examined in more detail.

In contrast to malocclusion, our study with harmonic occlusion using an occlusal bite splint (BS) indicates a tendency towards, but no significant improvement in maximum torque (*p* = 0.137), muscle performance (*p* > 0.999), and muscle work (*p* = 0.495). With the help of the T-Scan™-III (Tekscan Inc., South Boston, MA, USA) (36), we were able to induce harmonious tooth contacts on the BS so that we achieved a reproducible harmony with an occlusal right/left balance of 50%/50% ± 5% and were able to make comparable statements. The T-Scan™ technology enables occlusal forces to be measured objectively, accurately, and repeatedly during time measurement and to indicate the percentage difference between the right and left force components (37). A combination with marking foils is ideal because the pressure-sensitive foils in the T-Scan™-III system do not produce any contact markings intraorally. This combination enables the contacts depicted on the computer to be assigned intraorally with even greater precision (38). It is known from previous studies that the number of occlusal contacts and the function of the masticatory muscles are associated with higher muscle activity when the occlusion is more harmonious (39).

Despite this, further studies confirmed a minor influence of harmonic BS on isokinetic performance parameters in knee flexion and extension (23, 40). In isokinetic measurements of the upper body (shoulder abduction and adduction of the dominant arm), no significant effects on performance could be generated by wearing a harmonic BS (41). According to our methodology, the harmonic occlusion was individually ground in and harmonized on the subject. However, previous studies did not use any aid to confirm harmonic occlusion.

While the studies with isokinetic measurement methodology described above showed less influence of BS on performance parameters, studies with isometric measurement methodology showed significant advantages. Isometric examinations of the upper extremities in particular delivered significant results (42–44). This suggests that the type of measurement plays a crucial role (24).

Furthermore, the change in mandibular and temporomandibular joint position must be taken into consideration. In our study design, we did not manipulate the mandibular and temporomandibular joint (tmj) position in terms of a protrusive translational positioning of the condyles. The occlusion was first registered using an intraoral scanner in the maximum habitual intercuspidation (ICP).

Despite this, it needs to be taken into account that the bite elevation using BS is always associated with a change in the topographic condyle-fossa relationship and thus, with a new distribution of the contact surfaces of the articular surfaces (45).

Other studies investigated the effect of the position of the lower jaw on muscular performance parameters. Wearing a Mandibular Orthopedic Repositioning Appliance (MORA) significantly increased grip strength and EMG activity (46). The positive effect of a MORA was confirmed (42). When the mandibular condyles were positioned in a centric position, abduction and adduction of the shoulder and external/internal rotation of the arm increased peak torque and EMG activity (20). The myocentric (neuromuscular) tmj position increased maximal isometric force production and power in different jump types (47). Postural control and balance could also be improved by BS with mandibular positioning (48–50). In addition, decreases in muscle effort, lactic acid production, and heart rate were achieved (51).

However, there are also controversial results. In a study with an adjusted neuromuscular lower jaw position, no influence on dynamic balance could be measured (52). Sprint performance was also not improved (53).

Furthermore, it needs to be pointed out that different methods of bite registration were used. In some studies, the neurophysiological system is deprogrammed prior to functional mandibular positioning. This is done by biting on cotton rolls (44, 54), wearing an equalizer (50), or by prior TENS therapy (49, 52, 55). In other studies, the mandibular position is coded using a wax bite registration (48, 51) or in the articulator (20).

The effects of jaw clenching on the performance parameters have also been taken into consideration.

In a study by Chakfa et al. from 2002, a positive effect of a vertical change in occlusion by clenching on acrylic plates was demonstrated in isometric tests of arm abduction (43).

The increase in performance through jaw clenching is explained by the phenomenon of concurrent activation potentiation (CAP). This phenomenon is based on the remote voluntary contraction (RVC) of the temporomandibular system muscles during jaw clenching (16). During isometric contraction, performance parameters could be improved by clenching the jaws on an individualized splint. This condition was compared with clenching without a splint and also compared without occlusal contact (54). Maximum clenching on a vinyl mouthguard increased the rate of force development and reduced the time to peak force during the countermovement jump on a force platform (56). Positive effects were primarily demonstrated for isometric and static tests (16).

During the measurement runs, the subjects in our study were asked to bite on the splints. The comparatively better performance parameters with the harmonic splint could be explained by an increased CAP, as the bite surface is larger with the harmonic splint, allowing more masticatory force to be developed.

Lastly, the training status, homogeneity and sample size of the group of test subjects should be regarded. In our study design, we were able to include a very homogeneous group of well-trained competitive soccer athletes. All subjects had the same level of training, as they trained in the same club under identical training conditions. Nevertheless, it should be noted that the sample size (*n* = 30), which was determined by an *a priori* power analysis and considered sufficient, is relatively small, which limits the power of the results. Our results indicate a restricted positive influence of harmonious BS on performance parameters on trained competitive athletes. This result is confirmed by studies that showed no or little effect of BS and included only trained athletes (41, 52, 57). In contrast, studies with a positive effect of BS on muscle parameters were predominantly conducted with untrained subjects (20, 42–44, 49, 56).

We hypothesize that trained athletes already have a well-developed muscular and neurophysiological system that is less sensitive to changes in occlusion. The training status (trained vs. untrained) of the subjects could explain the discrepancy in the literature results. Further studies to investigate this hypothesis in a detailed fashion would be desirable.

As our study is a preliminary study, researchers should conduct further studies with a larger number of participants in which healthy subjects are compared with subjects with temperomandibular disorders. In this way, a better statement can be made about the effects of oral appliances on the entire body.

## 5 Conclusion

Our study showed that a harmonious occlusion by means of a harmonizing splint had no significant, but a tendency effect on the isokinetic parameters of the thigh muscles in young, well-trained soccer players. On the other hand, a simulated malocclusion led to significant performance losses. This indicates a potential importance of balanced occlusion for muscle performance, especially in terms of maximum strength, muscle work and muscle power.

The statistical relevance could be particularly relevant for sports where a splint is worn as a mouthguard. Based on our results, mouthguards should have a balanced and harmonious occlusion, as interfering contacts could impair athletic performance.

In addition, a holistic view of the body, including the stomatognathic system, should be considered when caring for professional athletes. It is advisable to involve a sports dentist in order to recognize and balance temperomandibular disorders or malocclusions at an early stage.

In view of the heterogeneous results in the literature, particularly with regard to the effect of occlusal splints in trained and untrained subjects, further studies are needed. Future studies should consider the differences between natural and simulated malocclusion, the training status of the subjects as well as different measurement methods and the effect of mandibular positioning approaches in order to better understand the underlying mechanisms and draw clearer conclusions.

## Data Availability

The raw data supporting the conclusions of this article will be made available by the authors, without undue reservation.
